# Congruent tactile stimulation reduces the strength of visual suppression during binocular rivalry

**DOI:** 10.1038/srep09413

**Published:** 2015-03-23

**Authors:** Claudia Lunghi, David Alais

**Affiliations:** 1 Department of Translational Research on New Technologies in Medicine and Surgery, University of Pisa, Pisa, Italy; 2 Institute of Neuroscience, CNR – Pisa, Via Moruzzi 1, 56124 Pisa, Italy; 3 School of Psychology, Brennan MacCallum Building, University of Sydney 2006, New South Wales, Australia

## Abstract

Presenting different images to each eye triggers ‘binocular rivalry’ in which one image is visible and the other suppressed, with the visible image alternating every second or so. We previously showed that binocular rivalry between cross-oriented gratings is altered when the fingertip explores a grooved stimulus aligned with one of the rivaling gratings: the matching visual grating's dominance duration was lengthened and its suppression duration shortened. In a more robust test, we here measure visual contrast sensitivity during rivalry dominance and suppression, with and without exploration of the grooved surface, to determine if rivalry suppression strength is modulated by touch. We find that a visual grating undergoes 45% less suppression when observers touch an aligned grating, compared to a cross-oriented one. Touching an aligned grating also improved visual detection thresholds for the ‘invisible’ suppressed grating by 2.4 dB, relative to a vision-only condition. These results show that congruent haptic stimulation prevents a visual stimulus from becoming deeply suppressed in binocular rivalry. Moreover, because congruent touch acted on the phenomenally invisible grating, this visuo-haptic interaction must precede awareness and likely occurs early in visual processing.

Recent work in the field of multisensory research has shown that cross-sensory interactions in the brain are extensive^1^,^2^,^3^ and may occur much earlier than previously thought, even between primary cortical areas^4^,^5^. Neurons in the early visual area V4 of rhesus monkeys have been shown to respond to tactile orientation stimulation^6^,^7^. A good deal of psychophysical evidence supports the view that basic auditory and visual stimuli interact^8^,^9^,^10^ and accumulating evidence suggests that vision and touch may also be functionally linked at early stages of processing^11^,^12^,^13^. Arabzedah, Clifford & Harris^14^ examined tactile intensity discrimination and found that an accompanying visual stimulus adjacent to the stimulated finger improved tactile sensitivity in way that was well modeled as a visual-tactile summation combining to elicit a stronger tactile response. Another study^15^ showed evidence of visual-tactile interactions using oriented grating stimuli, finding that detection of a visual grating was improved when exploring a congruent (i.e., same location and orientation) tactile grating. The relative orientation of the gratings was varied unpredictably between parallel and a range of non-parallel orientations and revealed a clear orientation tuning: tactile facilitation of visual detection only occurred when the visual and tactile gratings had the same orientation. The improvement in visual contrast sensitivity reported in this study may be due to convergence of visual and tactile feed-forward signals in early cortical areas where orientation selectivity is strong, effectively boosting the visual signal's strength.

One useful approach for revealing tactile influences on vision is to add tactile signals while the visual system is confronted with ambiguous visual inputs and unable to resolve a stable percept^16^,^17^. A standard way to induce perceptual ambiguity in vision is by using binocular rivalry^18^,^19^,^20^, the name given to the perceptual bistability that arises when each eye is presented with a different image. The incompatible monocular images prevent binocular fusion and trigger an irregular series of perceptual oscillations in which one image is perceived and then the other, in an ongoing stochastic alternation^18^,^19^,^20^. A number of studies have shown that tactile signals congruent with one of the competing visual images can help stabilize the perceptual ambiguity by biasing the alternation dynamics in favour of the crossmodally congruent stimulus^21^,^22^,^23^. In one study^22^, two visual gratings with orthogonal orientations were presented to each eye to produce binocular rivalry and participants continuously monitored the perceptual fluctuations between one grating and the other. Subjects intermittently explored for several seconds a tactile grating which was congruent with one of the visual stimuli. Tactile exploration extended the dominance duration of the matching visual stimulus (if it was currently dominant), or shortened its suppression duration when it was suppressed so that visual perception quickly changed to match the tactile stimulus. Subsequently, Lunghi and Alais^21^ showed this visual-haptic interaction in rivalry is tightly orientation tuned, with the effect declining rapidly as the visual-tactile orientation difference increases.

The exploration of crossmodal influences on binocular rivalry is informative for several reasons. One is that with visual perception unresolved and bistable, the role of crossmodal signals is more evident as even a relatively small input to vision can tilt the balance in favour of the congruent image and thus stabilize visual perception. Another reason is that the alternating monocular suppression that underlies the perceptual alternations in binocular rivalry is thought to occur early in visual processing where left- and right-eye signals are first combined^24^,^25^. Any crossmodal influence on rivalry therefore implies an early interaction. Consistent with this argument are results showing that the tactile interaction with rivalry is spatially localized and spatial-frequency tuned^22^,^26^ as well as orientation tuned^21^, reflecting the characteristics of neurons in early visual cortex. A final point of interest is that the tactile influence on binocular rivalry operates on both the dominant and the suppressed percept. This is significant because most modulatory effects on rivalry dynamics operate by extending the duration of the consciously perceived image and do not influence the competing percept that is suppressed from awareness. That a congruent tactile stimulus will rescue the matching visual stimulus from suppression^21^,^22^,^26^,^27^ also suggests an early visual-tactile interaction as it must interact with a stimulus that is thought to be suppressed early in visual processing and at a level preceding conscious awareness.

Here we extend the recent work on tactile influences on binocular rivalry by measuring whether visual contrast thresholds in rivalry are altered by congruent tactile input. Measuring contrast sensitivity for suppressed stimuli is widely done in rivalry research as it can be compared with sensitivity during dominance to obtain a measure of suppression strength known as suppression depth. Typically, the loss in contrast sensitivity during rivalry suppression is in the range of 0.3 to 0.5 log units relative to dominance sensitivity. Given the findings suggesting that congruent tactile input acts on the suppressed visual image and that tactile and visual signals can combine to boost signal strength^14^,^15^, we test the prediction that the visual grating undergoing suppression will maintain higher contrast sensitivity when it is paired with a congruent tactile grating than when paired with an incongruent one.

## Methods

### Ethics Statement

Participants gave written informed consent. The experimental procedure conformed to the declaration of Helsinki and was approved by the local ethics committee (Human Research Ethics Committee (HREC) Low Risk Executive Committee, University of Sydney, Protocol #14893).

### Subjects

Eight subjects (two males, mean age 30 ± 6 years), including the authors, took part in the experiment. All had normal or corrected-to-normal vision, good stereopsis and no strong eye dominance. All subjects (except the authors) were naïve to the purposes of the experiment.

### Apparatus and Stimuli

The experiment took place in a dark and quiet room. The visual stimuli were created in Matlab using the Psychtoolbox, displayed on a linearized 17″ CRT monitor (Mitsubishi Diamond Digital 771A, 800 × 600 pixels × 100 Hz) and viewed through a mirror stereoscope from a distance of 45 cm. The visual stimuli were orthogonal Gabor gratings (orientation ±45°, size 3°, S.F. 2 cpd, contrast 55%) presented in central vision on a uniform grey background (50.9 cd/m^2^) surrounded by a white fixation square to facilitate stable binocular fusion. A small, white, central fixation cross helped stabilize fixation and eye-movements. The contrast probe increment ranged from 10 to 81% of the grating contrast, each probe was presented for 250 ms with a Gaussian profile ramp of 70 ms to avoid a visual transient that could interfere with the dynamics of binocular rivalry, the contrast increment probe covered the whole upper or lower half of the stimulus. At each probe presentation, the contrast increment was randomly determined. At each experimental block the contrast increment probes were presented at a random interval (ranging from 1.5 to 3 s) on one of the two rivaling gratings. The orientation and the eye of presentation of the probed stimulus were varied at each experimental block and randomized for each observer. The haptic stimulus was a sinusoidal grating (size: 3 cm, spatial frequency 2 cyc/cm) created with a 3D printer and attached on a shaft on the bottom frame of the monitor, both the haptic grating and the shaft were hidden by a box, so that observers could not see neither the grating or their hand touching it. Although the visual and haptic stimuli were not collocated they were aligned horizontally and a spatial proximity illusion was created by the dark, open loop conditions in which observers could not see their hand or the haptic stimulus. The plane of the haptic stimulus was oriented vertically, parallel to the plane of the monitor. The efficacy of this arrangement has been demonstrated in previous experiments^21^,^22^. The orientation of the haptic stimulus (±45°) was changed at each experimental block and was either parallel or orthogonal to the visual stimulus that was probed.

### Experimental Procedure

To measure suppression depth we added brief contrast increments to a given eye's stimulus (with eye and stimulus both counterbalanced over observers), randomly in either the upper or lower half. In a two-alternative, forced-choice task, the observer indicated which half contained the increment. In typical suppression depth studies^28^,^29^, observers trigger the probe stimulus when they judge that they are in a fully suppressed (or dominant, depending on condition) perceptual state – effectively a dual-task paradigm that requires them to monitor their perceptual states and to make the probe response. Here we use a new method of measuring suppression depth^30^ which simplifies the task to a single response about probe location. In this new method, observers are not asked to track their rivalry states but by randomly sampling probes over a range of contrast over many trials a compound function of probe detection performance is obtained which can be decomposed into two separate psychometric functions, one for dominance and one for suppression^30^. The difference in the means of these distributions quantifies the magnitude of rivalry suppression.

**Figure 1:**
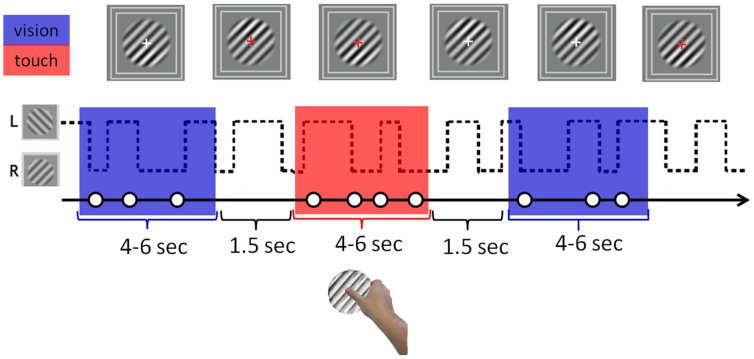
Experimental paradigm. Contrast increment probes of randomly varying intensity were presented either on the upper or lower half of one of the rivaling visual stimuli (orthogonal oblique gratings) at random intervals (1.5–3 s). A tone pip followed each probe presentation indicating to observers to report the location of the probe (upper or lower). Observers were not required to track their fluctuating perception during binocular rivalry and attended only to the contrast discrimination task. A change in the fixation cross's color signaled the onset and offset of a touch period during which observers haptically explored a haptic grating that was rotated either parallel or orthogonal to the visual stimulus being probed. Periods of visual-only and visuo-haptic stimulation of random duration (4–6 s) were interleaved in time with a resting period of 1.5 s during which no probes were presented.

We used this new method to measure suppression depth in three conditions: congruent visuo-haptic, incongruent visuo-haptic, and visual-only. Observers participated in 12 experimental sessions, each one lasting about 15 minutes. During each session, 200 contrast increment probes were presented: 100 of these were presented during haptic stimulation (in separate blocks, either congruent or incongruent with the probed visual stimulus) and 100 were presented during visual-only stimulation (a diagram of the experimental paradigm is shown in Figure 1). A total of 600 trials per condition (touch parallel, touch orthogonal, vision parallel, vision orthogonal) were collected per each subject. Before starting the experiment, observers viewed 5 minutes of binocular rivalry between gratings of equal contrast and spatial frequency but orthogonal orientation (the same as used for the main experiment) in order to ascertain that they had normal rivalry dynamics. Observers who reported more than 15% of mixed percepts or showing more than 65% of total dominance of one eye over the other were excluded from the experiment. Observers sat in front of the video monitor and rested their right hand close to the haptic stimulus located under a box which hid the hand and haptic stimulus from view. When the experimental session started, the rivaling visual gratings were presented and after each contrast-increment probe (which occurred randomly within the range of 2 to 3 s) a brief tone sounded which indicated to observers that a response was required to indicate whether they saw the probe on the top or the bottom part of the grating (2AFC task), guessing if necessary. Upon a change in color of the fixation cross (from white to red) observers were instructed to explore the haptic stimulus with the right thumb performing circular movements until the fixation cross changed in color again (from red to white). Observers were instructed that the haptic stimulus was not relevant to the task. Touch and no-touch periods (each one ranging from 4 to 6 s) were interleaved throughout the experimental session with a 1.5 s rest between them in which no probes were presented. This probe-free period allowed observers time to either reach for or release the haptic stimulus and was long enough to ensure that the non-touch periods were well segregated from touch periods. Moreover, this 1.5 rest period allowed rivalry dynamics to settle after haptic stimulation to minimize the possibility of haptic stimulation altering binocular rivalry dynamics. In debriefing sessions, observers reported perceiving the typical perceptual alternation characterizing the dynamics of binocular rivalry regardless of touch or no-touch conditions.

Two extra experimental sessions were acquired for each observer in which rivalry alternation dynamics were recorded. In these sessions, the probe stimuli were presented but participants were instructed to ignore them and to report their visual perception continuously by pressing appropriate keys on the keyboard with their left hand. These sessions enabled estimates of the proportion of contrast increment probes presented during dominance and suppression for each condition (visual-only and visuo-haptic stimulation), which in turn are used as weights in the model shown below (see Equation 1). Specifically, in order to obtain the weights, we asked observers to continuously report their visual perception although we only recorded the percept reported at the time of each probe presentation. Note that this step may not be necessary given equal stimulus contrasts in each eye and interocular counterbalancing to control for differences in ocular dominance, as weights of 0.5 could be justifiably assumed. Also, the rivalry dynamics could have differed slightly for the two tasks because of different attentional loads (asking observers to report rivalrous perception may have slowed switching rate^31^, however this would affect overall switching rate rather than one of the stimuli selectively, and thus would not change the dominance/suppression proportion).

### Analyses

We analysed discrimination performance for contrast increment probes using the new method recently described^30^ to define suppression depth for the three conditions (congruent visuo-haptic, incongruent visuo-haptic, and visual-only). The new method does not require subjects to track their alternating rivalry percepts but, with the assumption that alternations do occur, the discrimination curve must be a compound of dominance and suppression curves. The probe discrimination data can then be fitted with an ‘average of two psychometric functions’ model as shown below in Equation 1, allowing the separate psychometric functions for probe performance during dominance and during suppression phases of rivalry to be recovered. Let *c* be log_10_ contrast, *P*, the probability of a correct probe response, *G_D_* the psychometric function fitted as the lower (dominance) component, and *G_S_* the psychometric function fitted as the upper (suppression) component. The model fitted to the rivalry data is then:
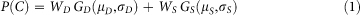
where
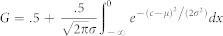


and the fitted parameters are:
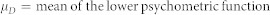

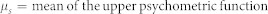

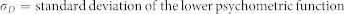

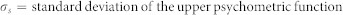


The weights of each psychometric function are determined by measuring the relative predominance of the rivalry stimuli.
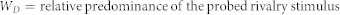

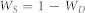


Separate predominance measures were made for the visual-only and visuo-haptic conditions measured in the two extra experimental sessions.

**Figure 2:**
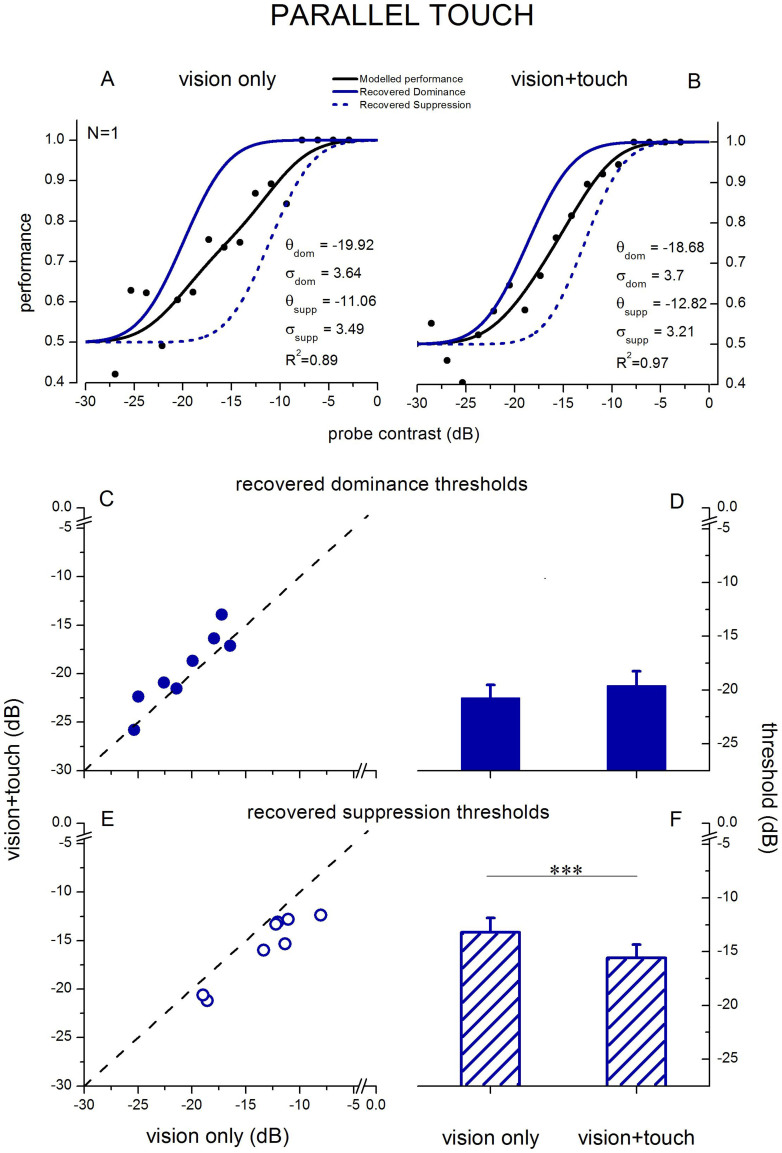
Effect of parallel haptic stimulation on contrast increment thresholds during binocular rivalry dominance and suppression. Psychometric functions plotting probe detection performance as a function of the probe's contrast increment (black symbols) are shown for a single subject: (A) visual-only condition, (B) parallel visuo-haptic condition (red lines). The black functions show performance obtained during rivalry without observers indicating their fluctuating perceptual states. A new model^30^, as shown in Equation 1, was used to decompose the compound function shown in black to recover the component psychometric functions for dominance (solid lines) and suppression (dashed lines) and their threshold (μ) and slope (σ) parameters. Average and single subjects' contrast discrimination thresholds measured during dominance (C, D) and suppression (E, F): parallel visuo-haptic stimulation improved contrast discrimination sensitivity during binocular rivalry suppression (*** represents p < 0.001). Error bars represent s.e.m.

**Figure 3:**
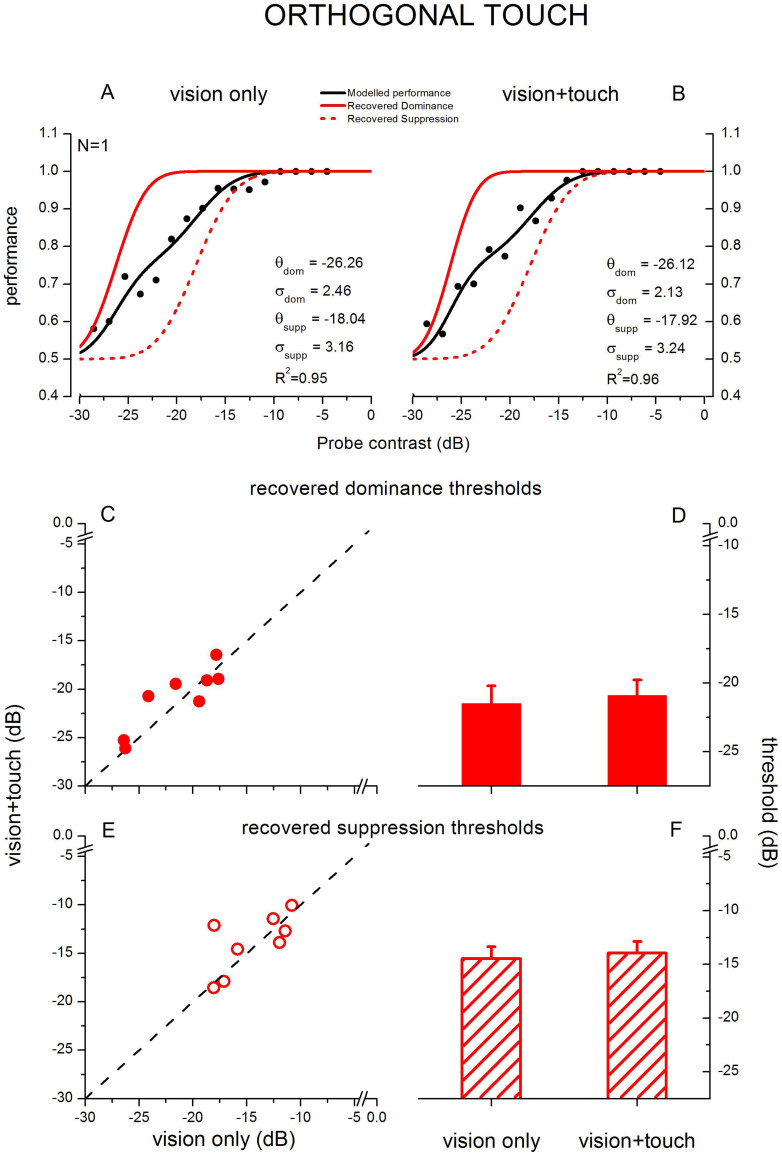
Effect orthogonal haptic stimulation on contrast increment thresholds during binocular rivalry dominance and suppression. Upper panels show psychometric functions plotting probe detection performance as a function of the probe's contrast increment (black symbols), pooled across all observers. These functions are obtained during rivalry without observers indicating their fluctuating perceptual states. A new model^30^, as shown in Equation 1, was used to recover the psychometric functions for the dominance (solid lines) and suppression (dashed lines) components of the compound function shown in black. The results for the visual-only condition are shown in A and for the orthogonal visuo-haptic condition in B). Average and single subjects' contrast discrimination thresholds measured during dominance (C, D) and suppression (E, F): orthogonal visuo-haptic stimulation did not alter discrimination sensitivity during binocular rivalry suppression. Error bars represent s.e.m.

The data from each individual observer were analysed separately. This involved pooling responses to the randomly varying contrast probe into a series of narrow bins (optimal bin-width was computed individually for each observer, ranging from 1.35 to 1.65 dB, in order to achieve the best model fit) to calculate mean performance as a function of contrast (see binned data in Figure 2A–B and Figure 3A–B). In fact, the average R^2^ for visual only stimulation were 0.88 ± 0.03 and.91 ± 0.03 and for visuo-haptic stimulation were.89 ± 0.03 and.96 ± 0.01, indicating excellent model fit. The model was fitted to the binned data and the component functions for dominance and suppression were recovered. The weightings of the component psychometric functions in the model were based on the relative proportion of contrast increment probes presented during dominance and during suppression for each observer, computed separately for each of the three conditions. Weights measured during visual-only and visuo-haptic stimulation were not statistically different from each other or from 0.5 (as expected, given equal contrasts and interocular counterbalancing), so the procedure of weighting the psychometric functions did not play a critical role in analyzing and interpreting the results and was potentially unnecessary. However, since haptic stimulation biases the dynamics of binocular rivalry^21^,^22^,^26^,^27^, we measured the weights to be sure that our effect on binocular rivalry suppression depth was not due to a change in alternation dynamics (e.g., to a bias causing more contrast probes to be presented during dominance in the parallel visuo-haptic condition). The equally balanced weights we obtained are likely due to several factors: equal contrasts, interocular counterbalancing and a 1.5 s probe-free period before and after each touch period to allow rivalry dynamics to settle. The strength of rivalry suppression (in dB contrast) is defined as the threshold (μ) of the suppression function minus the threshold of the dominance function. Suppression depth and thresholds were compared at the group-level using paired-sample t-tests, repeated measures ANOVA and a non-parametric permutation test.

## Results

We obtained thresholds for probe discrimination in dominance and suppression using the model shown in Equation 1, comparing performance in visual-only stimulation with performance in congruent visual-haptic (parallel orientation) stimulation and in incongruent visual-haptic (orthogonal orientation) stimulation (a diagram of the experimental paradigm is reported in Figure 1). Figure 2A–B and Figure 3A–B show modeled psychometric functions for one observer. Taking the congruent haptic condition first, we found that during congruent haptic stimulation, contrast increment detection thresholds measured during binocular rivalry suppression improved significantly compared to visual-only stimulation (paired samples t-test, t(7) = 5.47, α = 0.05, p < 0.001, permutation test based on t-test: p < 0.001 Figure 2F,). Interestingly, the improvement of ~2.4 dB (average contrast increment detection threshold for visual only stimulation: −13.2 ± 1.33 dB, parallel visuo-haptic stimulation: −15.59 ± 1.24 dB) was observed only for suppression (the effect is clear from inspection of figure 2D in which individual subjects' thresholds for parallel visuo-haptic and visual only stimulation are plotted against each other: all points lie beneath the unity line indicating lower thresholds for parallel visuo-haptic stimulation), as thresholds measured during dominance did not statistically differ between visual-only and congruent haptic stimulation (in figure 2B the symbols representing individual subjects' thresholds are scattered around the unity line, paired samples t-test, t(7) = 2.24, α = 0.05, p = 0.06, permutation test: p = 0.32). A two-way repeated-measures ANOVA revealed a significant effect of the rivalry state factor (dominance vs. suppression, F(1,7) = 76.88, p < 0.001), as expected. An effect of the type of stimulation was not significant (visual-only vs. visuo-haptic stimulation, F(1,7) = 1.88, p = 0.21), and the interaction between the two factors was highly significant (F(1,7) = 139.74, p < 0.001). When running a Bonferroni test on pairwise comparison, the difference between suppression detection thresholds measured during visual-only and visuo-haptic stimulation remained significant (t(5) = 2.4, p = 0.05).

It is notable that the slopes of the psychometric functions were comparable in all conditions. This indicates that observers' precision for making the visual judgment about probe location was not affected by whether the condition was visual-only or visuo-haptic. Tests on the slopes for recovered dominance during visual-only stimulation, 3.32 ± 0.66 dB, and parallel visuo-haptic stimulation. 4.7 ± 0.5 dB, did not differ: paired samples t-test, t(7) = 1.57, α = 0.05, p = 0.16, permutation test: p = 0.06. Slopes for recovered suppression during visual-only stimulation, 3.39 ± 0.49 dB, and parallel visuo-haptic stimulation, 4.13 ± 0.49 dB, did not differ: paired samples t-test, t(7) = 1.27, α = 0.05, p = 0.24, permutation test: p = 0.21), indicating that the dual task in the haptic conditions (exploring the haptic grating in addition to reporting the probe location) did not interfere with observers' precision in the visual task. These results are confirmed in both the group mean data (Figure 2D–F) and in analyses of individual observers (Figure 2A–B–C–E).

In the incongruent haptic condition, where the haptic grating was oriented orthogonally to the probed visual stimulus (Figure 3), haptic stimulation had no effect on probe discrimination thresholds. Dominance thresholds were very similar in both the visual-only and visual-haptic conditions, as they were for suppression. A two-way repeated-measures ANOVA revealed a significant effect of the viewing condition factor (dominance and suppression, F(1,7) = 229.06, p < 0.001), which reflects the expected decrease of contrast sensitivity provoked by binocular rivalry suppression, but no effect of the type of stimulation factor (visual-only vs. visuo-haptic, F(1,7) = 0.75, p < 0.41) and no interaction between the two factors (F(1,7) = 0.01, p < 0.93). Again, there was no difference between the slopes of the psychometric functions in all conditions (Slopes for recovered dominance during visual only stimulation, 3.31 ± 0.46 dB, and orthogonal visuo-haptic stimulation, 3.07 ± 0.34 dB, did not differ: paired samples t-test, t(7) = 0.96, α = 0.05, p = 0.37, permutation test: p = 0.82. Slopes for recovered suppression during visual-only stimulation, 3.8 ± 0.53 dB, and parallel visuo-haptic stimulation, 3.54 ± 0.34 dB, did not differ: paired samples t-test, t(7) = 0.82, α = 0.05, p = 0.44, permutation test: p = 0.91). These results confirm that performing a second task in the visual-haptic condition (i.e., exploring the tactile grating) did not interfere with performance on the visual task.

**Figure 4:**
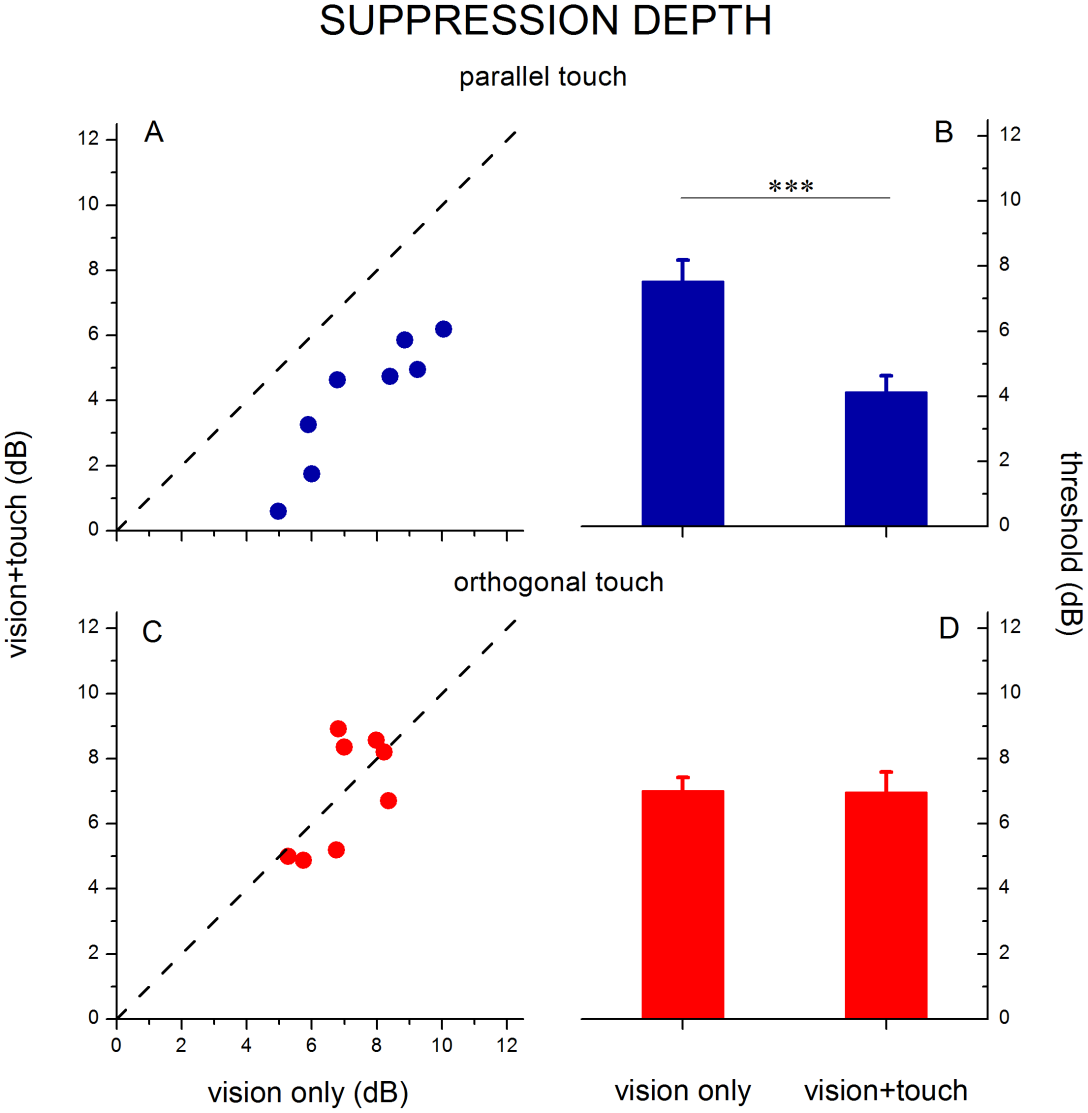
Binocular rivalry suppression depth. Suppression depth, defined as the difference between contrast increment thresholds measured during dominance and during suppression, are plotted for parallel (single subjects A, average B) and orthogonal (single subjects C, average D) visuo-haptic stimulation and compared to the visual-only condition. During parallel visuo-haptic stimulation, suppression depth decreases by about a factor of 2 compared with visual only stimulation (*** represents p < 0.001). When the haptic stimulation was orthogonal to the probed visual stimulus, no difference in suppression depth was found relative to visual-only stimulation. Error bars represent s.e.m.

In order to compare the strength of suppression during binocular rivalry between the visual-only condition and the two different types of visual-haptic stimulation, we computed *suppression depth*. Suppression depth is defined as the difference between the detection threshold measured during suppression and that measured during dominance. Suppression depth was calculated for each of the three conditions and is shown in Figure 4. The strength of suppression was very similar in the two visual-only conditions, as expected, as these were identical conditions (one interleaved with the congruent haptic condition and the other with the incongruent condition), however, since visual-only and visuo-haptic stimulations were interleaved within experimental blocks, we decided to maintain the two visual-only conditions separated. Strikingly, in the congruent haptic condition, adding the parallel haptic stimulus caused suppression strength to decline by a factor of 1.9, from 7.53 ± 0.65 dB to 3.99 ± 0.7 dB. This decline in suppression strength was significant (t(7) = 11.96, α = 0.05, p < 0.001, permutation test: p < 0.001). Indeed, the decline in suppression strength for congruent haptic stimulation was significant compared to the incongruent condition (t(7) = 3.63, α = 0.05, p < 0.008), showing that the haptic effect on rivalry suppression is dependent on congruent orientation. A two-way repeated-measures ANOVA revealed an effect of the type of stimulation (visual-only vs. visuo-haptic), F(1,7) = 31.42, p < 0.001, and a significant interaction between the type of stimulation and visuo-haptic orientation (parallel vs. orthogonal), F(1,7) = 56.46, p < 0.001). Moreover, the significance of the difference between suppression depth measured during visual-only and congruent visuo-haptic stimulation remained significant in a Bonferroni-corrected pairwise comparison (t(7) = 4.9, p = 0.011), as well as the difference between suppression depth measured during parallel and orthogonal visuo-haptic stimulation (t(7) = 4.13, p = 0.027).

## Discussion

We measured the strength of binocular rivalry suppression for rivaling orthogonal gratings with and without haptic stimuli. The haptic stimuli were gratings located adjacent to the visual gratings and had the same size and spatial frequency as the visual stimuli. Our central finding is that haptic exploration of a grating matching the orientation of the probed visual stimulus greatly attenuates binocular rivalry suppression, reducing suppression strength by a factor of 2. Suppression strength in the visual-only condition averaged ~7.5 dB and was reduced to ~4 dB in the congruent haptic condition. The effect manifests by improving visual contrast sensitivity in the suppressed eye rather than altering sensitivity in the dominant eye, meaning that although the suppressed visual stimulus is attenuated beneath the threshold for awareness, it is not as deeply suppressed when it is bound with a congruent tactile stimulus. Congruency between the visual and haptic stimuli is critical in this effect because a haptic stimulus oriented orthogonally to the probed visual stimulus failed to significantly reduce rivalry suppression strength. Our results imply that the visuo-haptic interaction occurs early in visual processing for three reasons. First, the haptic stimulus combined with the visual stimulus that was phenomenally suppressed, suggesting an interaction occurring prior to awareness. Second, the effect was orientation selective, as haptic stimulation orthogonal to the probed visual grating did not reduce the depth of suppression. Third, the effect of haptic input was to improve contrast sensitivity – a fundamental attribute in early visual cortex – by shifting the psychometric function to the left.

Our results are in agreement with a previous study^15^ showing in a two-alternative, forced-choice task that congruent haptic stimulation improves visual contrast sensitivity for detection of visual gratings embedded in noise. Interestingly, these authors also showed that the visual facilitation induced by haptic exploration of a tactile grating was strictly tuned for matched visuo-haptic orientation. Although we did not measure the orientation tuning of the effect reported here, the tight orientation tuning these authors reported for visuo-haptic interaction was similar to the narrow orientation tuning reported in another binocular rivalry study which explored visuo-haptic interactions among grating stimuli^21^. Another interesting aspect of this non-rivalry visual-haptic study was that exploring a haptic grating orthogonal to the visual grating impaired visual contrast detection, suggesting a cross-modal form of cross-orientation inhibition^15^. However, in that study^15^, visual contrast sensitivity was not measured in the absence of haptic stimulation as comparisons were always between visual contrast thresholds acquired with congruent (parallel) or incongruent (orthogonal) haptic stimulation. Our results reported here confirm that haptic stimulation affects visual contrast sensitivity, but by measuring contrast thresholds also during visual-only stimulation and, more importantly in the absence of visual awareness during binocular rivalry suppression, we both extend the results of van der Groen et al^15^ and effectively rule out the possibility of response bias.

The results presented here help clarify the mechanisms underlying a series of visuo-haptic interactions recently reported in binocular rivalry^21^,^22^,^26^,^27^. By quantifying visual contrast sensitivity in both dominance and suppression states of rivalry, our results demonstrate clearly that congruent haptic stimulation influences vision during binocular rivalry mainly by preventing the suppressed visual stimulus from becoming deeply suppressed. This is important as previous studies measured how haptic stimuli influenced binocular rivalry alternation dynamics, finding that the likelihood of perceiving a given visual orientation was higher when observers explored a matching haptic stimulus. Such an approach is open to response bias and demand characteristics as the reported visual orientation in binocular rivalry is purely subjective and unverifiable. By using a new and more objective method to measure binocular rivalry suppression depth which does not require observers to report their subjective fluctuations in perceptual state, as recently proposed by Alais et al^30^, we can rule out the possibility of response bias playing an important role in driving the haptic effect on rivalry suppression. In our study observers did not report their rivalry alternations and only performed the contrast discrimination task, making it implausible that observers overtly favored the visual stimulus parallel to the haptic one. This single-task approach provides a better measure of visual contrast sensitivity during rivalry compared to the dual-task approach requiring subjects to simultaneously report their fluctuating perceptual states while also making visual contrast discriminations, and still allows a separation of performance during dominance and suppression states to be recovered in a subsequent off-line analysis^30^.

Evidence suggests that binocular rivalry suppression occurs early in the visual system. One line of evidence for an early locus of rivalry suppression comes from neuroimaging studies showing activity in V1 correlates with rivalry perception^24^,^25^. Another comes from psychophysical adaptation studies showing that only basic adaptation aftereffects survive binocular rivalry suppression and continue to build in magnitude even when rendered invisible by periods of rivalry suppression. Examples include the tilt aftereffect^32^, the spatial frequency and threshold elevation aftereffects^33^, the orientation-contingent colour aftereffect and the translational motion aftereffect^28^,^32^,^33^,^34^,^35^. More complex aftereffects such as motion aftereffects for spiral motion, plaid motion and optic flow are disrupted by binocular rivalry suppression and show a reduced strength compared to non-rivalry conditions^36^,^37^,^38^,^39^. Importantly, aftereffects that survive binocular rivalry suppression are thought to originate from neural adaptation entirely^40^,^41^,^42^,^43^,^44^,^45^, or at least in part^46^,^47^, in early visual cortices (V1–V2), suggesting that binocular rivalry suppression begins at the level of primary visual cortex, although may continue to increase in subsequent stages of visual processing^29^. Our finding that haptic stimulation increases visual contrast sensitivity during suppression therefore reinforces the hypothesis that the interaction between visual and haptic signals observed during binocular rivalry occurs early in the visual system, probably already in the primary visual cortex.

Evidence from other sources also supports the idea that haptic signals input to primary visual cortex. Transcranial stimulation of visual cortical areas has been also shown to improve tactile discrimination^48^,^49^, pointing to a role of visual cortex in contributing to tactile processing. Recruitment of V1 has been shown after prolonged practice on a tactile task in expert Mah-Jong players^50^ and tactile stimulation modulates BOLD activity (both activation and deactivation) in early visual areas^51^. Importantly, the primary visual cortex of early-blind subjects is activated during auditory^52^, tactile^53^,^54^ and verbal tasks^55^,^56^, indicating cross-modal plasticity following sensory loss. The interesting question is how this plasticity occurs: are the connections between auditory and somatosensory areas to V1 created *ex novo* or are there pre-existing (sparse) connections that become reinforced following sensory loss? Kauffman et al^57^ found tactile performance on a Braille-character discrimination task was significantly improved in normal sighted individuals after five days of blindfolding^57^. A subsequent experiment showed this advance in tactile discrimination was accompanied by increased BOLD activation in primary visual cortex during tactile stimulation^58^. Interestingly, inhibiting occipital cortex with repeated transcranial stimulation at 1 Hz annulled the improvement in tactile discrimination gained by the blindfolded individuals, suggesting a functional role for early visual cortex recruitment^58^. These studies support the hypothesis that cross-modal plasticity in blind patients involving V1 recruitment results from reinforcement of pre-existing connections. We suggest that such connections mediate the haptically increased visual sensitivity we observed during binocular rivalry suppression. Being sparse, such connections in normally sighted individuals would be largely masked by dominant visual input. We propose that when visual sensitivity is attenuated by rivalry suppression, the relative weight of haptic input is increased and produces enhanced detection of the visual grating when the haptic input is congruent. By contrast, during rivalry dominance, visual function is optimal and the relative weight of haptic input is too weak to produce an improvement in contrast sensitivity.

In sum, we have shown that oriented haptic stimulation prevents a congruently oriented visual stimulus from becoming deeply suppressed in binocular rivalry. This implies a visuo-haptic interaction occurring early in visual processing prior to awareness and supports emerging evidence of direct connectivity between early unisensory cortices^3^,^4^,^5^,^59^. The interaction is orientation selective, consistent with recent visuo-tactile findings^15^,^21^,^22^,^26^ and with recent work highlighting orientation processing in the tactile domain^60^.

## Author Contributions

C.L. and D.A. designed research, D.A. programmed the visual stimuli and designed the model, C.L. collected and analyzed the data, C.L. and D.A. wrote the paper.
